# Safety analysis of Oseltamivir and Baloxavir Marboxil after market approval: a pharmacovigilance study based on the FDA adverse event reporting system

**DOI:** 10.1186/s12879-024-09339-4

**Published:** 2024-05-09

**Authors:** Yunsong Li, Xiaoling Wang, Yufang Liao, Yanbin Zeng, Wanlong Lin, Wei Zhuang

**Affiliations:** Department of Pharmacy, Women and Children’s Hospital, School of Medicine, Xiamen University, 10# Zhenhai Road, Xiamen, China

**Keywords:** Oseltamivir, Baloxavir Marboxil, Fulminant hepatitis, Rhabdomyolysis, Safety, Side effect

## Abstract

**Background and objectives:**

Amidst limited influenza treatment options, evaluating the safety of Oseltamivir and Baloxavir Marboxil is crucial, particularly given their comparable efficacy. This study investigates post-market safety profiles, exploring adverse events (AEs) and their drug associations to provide essential clinical references.

**Methods:**

A meticulous analysis of FDA Adverse Event Reporting System (FAERS) data spanning the first quarter of 2004 to the fourth quarter of 2022 was conducted. Using data mining techniques like reporting odds ratio (ROR), proportional reporting ratio, Bayesian Confidence Propagation Neural Network, and Multiple Gamma Poisson Shrinkage, AEs related to Oseltamivir and Baloxavir Marboxil were examined. Venn analysis compared and selected specific AEs associated with each drug.

**Results:**

Incorporating 15,104 Oseltamivir cases and 1,594 Baloxavir Marboxil cases, Wain analysis unveiled 21 common AEs across neurological, psychiatric, gastrointestinal, dermatological, respiratory, and infectious domains. Oseltamivir exhibited 221 significantly specific AEs, including appendicolith [ROR (95% CI), 459.53 (340.88 ∼ 619.47)], acne infantile [ROR (95% CI, 368.65 (118.89 ∼ 1143.09)], acute macular neuroretinopathy [ROR (95% CI), 294.92 (97.88 ∼ 888.64)], proctitis [ROR (95% CI), 245.74 (101.47 ∼ 595.31)], and Purpura senile [ROR (95% CI), 154.02 (81.96 ∼ 289.43)]. designated adverse events (DMEs) associated with Oseltamivir included fulminant hepatitis [ROR (95% CI), 12.12 (8.30-17.72), *n*=27], ventricular fibrillation [ROR (95% CI), 7.68 (6.01–9.83), n=64], toxic epidermal necrolysis [ROR (95% CI), 7.21 (5.74–9.05), n=75]. Baloxavir Marboxil exhibited 34 specific AEs, including Melaena [ROR (95% CI), 21.34 (14.15–32.18), *n* = 23], cystitis haemorrhagic [ROR (95% CI), 20.22 (7.57-54.00), *n* = 4], ileus paralytic [ROR (95% CI), 18.57 (5.98–57.71), *n* = 3], and haemorrhagic diathesis [ROR (95% CI), 16.86 (5.43–52.40)), *n* = 3]. DMEs associated with Baloxavir Marboxil included rhabdomyolysis [ROR (95% CI), 15.50 (10.53 ∼ 22.80), *n* = 26].

**Conclusion:**

Monitoring fulminant hepatitis during Oseltamivir treatment, especially in patients with liver-related diseases, is crucial. Oseltamivir’s potential to induce abnormal behavior, especially in adolescents, necessitates special attention. Baloxavir Marboxil, with lower hepatic toxicity, emerges as a potential alternative for patients with liver diseases. During Baloxavir Marboxil treatment, focused attention on the occurrence of rhabdomyolysis is advised, necessitating timely monitoring of relevant indicators for those with clinical manifestations. The comprehensive data aims to provide valuable insights for clinicians and healthcare practitioners, facilitating an understanding of the safety profiles of these influenza treatments in real-world scenarios.

## Background

Seasonal influenza stands as one of the most prevalent viral respiratory diseases, with annual prevalence and mortality rates closely tied to global epidemics of influenza A and B viruses. A recent model study estimated a yearly global toll of approximately 290,000 to 650,000 respiratory disease-related deaths associated with seasonal influenza [1]. Neuraminidase inhibitors and RNA polymerase inhibitors constitute common interventions for influenza treatment and prevention. The Infectious Diseases Society of America guidelines advocate initiating antiviral treatment with a single neuraminidase inhibitor (NAI) such as oral oseltamivir, inhaled zanamivir, or intravenous peramivir as promptly as possible for suspected or confirmed influenza patients [2]. Studies indicate [3] no deaths in outpatient patients treated with oseltamivir and baloxavir marboxil. In hospitalized patients, baloxavir marboxil has demonstrated reduced mortality and significantly shortened hospital stays compared to oseltamivir. Moreover, in outpatient patients, the incidence of adverse events (AEs) with baloxavir marboxil was significantly lower than with oseltamivir. Literature has also reported [4] no apparent differences in safety between baloxavir and oseltamivir and other antiviral drugs. However, comprehensive safety studies between the two in a real-world setting with a large sample size remain unexplored.

Pharmacovigilance studies play a vital role in supplementing real-world drug use safety [5]. Spontaneous reporting systems, involving roles such as clinicians, pharmacists, patients, parents, and others, collect and record various drug-related AEs. These systems, particularly the FDA Adverse Event Reporting System (FAERS), serve as valuable resources for early detection and identification of potential adverse effects, facilitating continuous monitoring and tracking of AEs over time through data mining [6, 7]. By providing a readily available data source, spontaneous reporting databases contribute significantly to the timely identification of safety issues related to drug therapy in real-world environments [8]. The FAERS is one such spontaneous reporting system for collecting AEs. It is a readily available data source used to early identify safety issues related to drug therapy in the real world from a large population [7].

This study aims to analyze real-world safety data of oseltamivir and baloxavir marboxil using FAERS. It visualizes AE categories for both drugs, with a focus on designated adverse events (DME)s, providing insights into preferred treatment options for seasonal influenza from a safety perspective.

## Methods

### Data source

Data for this study were sourced from the FAERS, publicly available since 2004 and updated quarterly. Adverse reaction data for oseltamivir (from the first quarter of 2004 to the fourth quarter of 2022) and baloxavir marboxil (from the first quarter of 2018 to the fourth quarter of 2022) were downloaded and imported into SAS 9.4 for cleaning and analysis. The FAERS data include seven tables (DEMO, DRUG, REAC, OUTC, PRSR, INDI, and THER) linked by PrimaryID and CaseID.

### Data cleaning and standardization

As FAERS is a spontaneous reporting system, duplicate records were removed. Data from the DEMO table were selected, and duplicate records were eliminated based on CASEID, FDA_DT, and PRIMARYID. Analyses involving three or more reports were included. Excluded events included those labeled as “no adverse event,” “influenza,” “influenza pneumonia,” “influenza encephalitis,” “product problem,” “normal newborn,” “pregnancy,” and “various injuries, poisonings, and procedural complications.”

AEs were recorded using Preferred Terms (PT) from the Medical Dictionary for Drug Regulatory Activities (MedDRA). This study utilized hierarchical term sets of PTs and System Organ Class (SOC) terms for categorization and standardization.

### Data analysis

The Reporting Odd Ratio (ROR), ROR025 > 1,which is a lower limit of 95% confidence interval, as a detection criterion [8]; Proportional Reporting Ratio (PRR), there have been many reports by the signal detection criteria used in MHRA (PRR ≥ 2,χ2 ≥ 4, and *N* ≥ 3);Bayesian Confidence Propagation Neural Network (BCPNN), A no-information prior distribution is used as the prior distribution, and a signal is detected when the lower limit of the 95%credible interval of the IC(IC025) > 0;and Multi-Item Gamma Poisson Shrinker (MGPS), If EB05 ≥ 2, the AE can be interpreted as a signal [8]. Methods were employed for data mining (Table 1). detailed algorithms and formulas are available in (Table 2) In this study, the situation where all four methods have statistical significance is regarded as producing a safety signal, in order to reduce the generation of false positive signals. In the FAERS database, AEs may include disease symptoms and disease progression to reduce bias towards disease-related events. Venn analysis was used to screen common and drug-specific AEs. The analysis was performed using Venn’s online tool.

**Table 1:** Four grid table of proportional imbalance method

	Number of target adverse events	Number of other adverse events
Target Drug	a	b
Other Drugs	c	d

**Table 2:** Calculation formulas and detection standards of signal mining

Method	Computational formula	Threshold value
ROR	$\text{R}\text{O}\text{R}=\frac{(a/c)}{(b/d)}=\frac{ad}{bc}$ $95\text{\%}\text{C}\text{I}={\text{e}}^{\text{l}\text{n}\left(\text{R}\text{O}\text{R}\right)\pm 1.96\sqrt{ (\frac{1}{\text{a}}+\frac{1}{\text{b}}+\frac{1}{\text{c}}+\frac{1}{\text{d}}) }}$	ROR_025_ > 1, *N* ≥ 3
PRR	$\text{P}\text{R}\text{R}?\frac{a/(a+b)}{c/(c+d)}$ ${\chi }2=\frac{ {(\text{a}\text{d}-\text{b}\text{c})}^{2}(\text{a}+\text{b}+\text{c}+\text{d})}{( \text{a}+\text{b})(\text{a}+\text{c})(\text{c}+\text{d})(\text{b}+\text{d})}$	PRR ≥ 2, ${\chi }2\ge$4, *N*≥3
BCPNN	$\text{IC}={log}_{2}\frac{p(x,y)}{p\left(x\right)p\left(y\right)}={log}_{2}\frac{a(a+b+c+d)}{(a+b)(a+c)}$ $\text{I}\text{C}025={\text{e}}^{\text{l}\text{n}\left(\text{I}\text{C}\right)\pm 1.96\sqrt{ (\frac{1}{\text{a}}+\frac{1}{\text{b}}+\frac{1}{\text{c}}+\frac{1}{\text{d}}) }}$	IC_025_ > 0
MGPS	$\text{E}\text{B}\text{G}\text{M}?\frac{a(a+b+c+d)}{(a+c)(a+b)}$ $\text{E}\text{B}\text{G}\text{M}05={\text{e}}^{\text{l}\text{n}\left(\text{E}\text{B}\text{G}\text{M}\right)\pm 1.96\sqrt{ (\frac{1}{\text{a}}+\frac{1}{\text{b}}+\frac{1}{\text{c}}+\frac{1}{\text{d}}) }}$	EBGM_05_ > 2

## Results

### Characteristics of cases

As shown in Table 3, a total of 15,104 cases associated with Oseltamivir and 1,594 cases linked to Baloxavir Marboxil were identified. Within the 2,753 different types of AE reports for Oseltamivir, totaling 43,675 reports, 242 AEs displayed significant safety signals. In the 536 different types of AE reports for Baloxavir Marboxil, comprising 3,315 reports, 55 AEs exhibited significant safety signals. The fundamental characteristics of the cases are detailed in Table 3. The male-to-female ratios in AE reports for Oseltamivir and Baloxavir Marboxil were 1.32 and 1.16, respectively. Health professionals, including clinical doctors, nurses, or pharmacists, accounted for 23.57% of Oseltamivir reports and 65.75% of Baloxavir Marboxil reports. Both drugs displayed a concentration of reports in the United States and Japan. Age distribution in Oseltamivir reports primarily fell within the 18–64 age group, while Baloxavir Marboxil reports were predominantly below 64 years old, with a higher proportion in the under 18 age group.

**Table 3:** Basic information of Oseltamivir and Baloxavir Marboxil case reports (n%)

Project	Oseltamivir (*n* = 15,104)	Baloxavir Marboxil (*n* = 1594)
**Sex**		
Female	7462(49.40)	671(42.10)
Male	5660(37.47)	580(36.39)
NA	1982(13.12)	343(21.52)
**AEs Reports number**	43,675	3,315
**Reporters’ role**		
NA	5541(36.69)	1(0.06)
Consumer	4324(28.63)	545(34.19)
Lawyer	1680(11.12)	0(0.00)
Physician	1161(7.69)	624(39.15)
Other health-professional	2376(15.73)	213(13.36)
Pharmacist	22(0.15)	211(13.24)
**Reporters’ country**		
United States	6857(45.40)	916(57.47)
Japan	3114(20.63)	663(41.59)
Canada	453(3.00)	2(0.13)
Germany	162(1.07)	1(0.06)
Others	4673(30.94)	11(0.69)
NA	514(3.40)	1(0.06)
**Age group**		
< 18	3398(22.50)	421(26.41)
> 65	1830(12.12)	191(11.98)
18–64	4622(30.60)	383(24.03)
NA	5254(34.79)	599(37.58)
**Reported year**		
2004	142(0.94)	0
2005	363(2.40)	0
2006	252(1.67)	0
2007	541(3.58)	0
2008	348(2.30)	0
2009	1711(11.33)	0
2010	1318(8.73)	0
2011	472(3.13)	0
2012	396(2.62)	0
2013	519(3.44)	0
2014	374(2.48)	0
2015	1167(7.73)	0
2016	880(5.83)	0
2017	1730(11.45)	0
2018	1392(9.22)	10(0.63)
2019	999(6.61)	796(49.94)
2020	1222(8.09)	660(41.41)
2021	873(5.78)	49(3.07)
2022	405(2.68)	79(4.96)

### Age-based stratified analysis of System Organ Class (SOC) signals for Oseltamivir and Baloxavir Marboxil subgroups

**Fig. 1:**
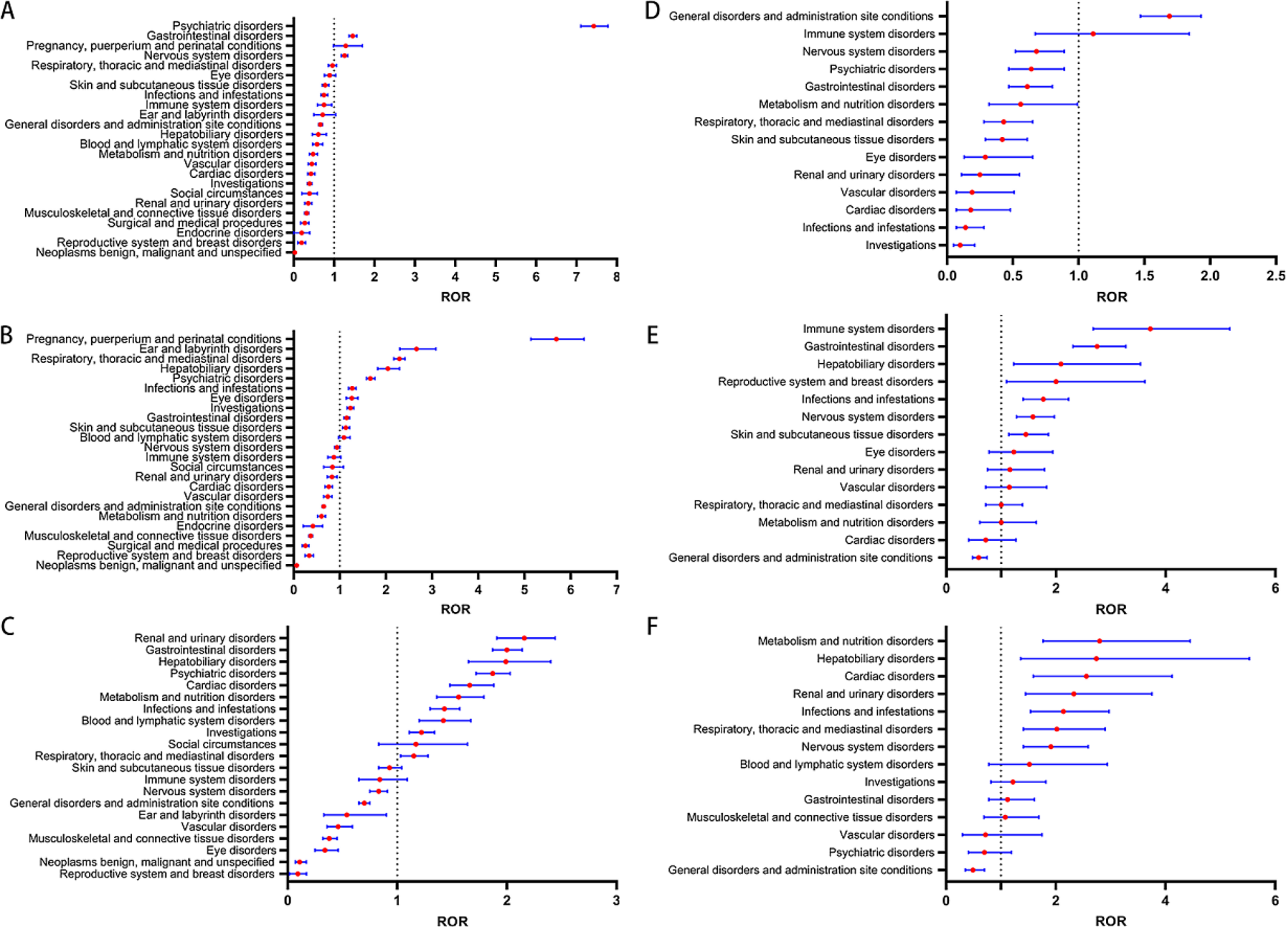
System Organ Class (SOC) Safety Signals in different age groups. Fig. **A-C** represent safety signals for patients treated with Oseltamivir, where (**A**) is for < 18 years, (**B**) is for 18–64 years, and (**C**) is for > 64 years. Fig. D-F represent safety signals for patients treated with Baloxavir Marboxil, where (**D**) is for < 18 years, (**E**) is for 18–64 years, and **(F**) is for > 64 years. SOC stands for System Organ Class, and ROR stands for Reporting Odds Ratio

Differential analysis of SOC signal strength was performed for age groups < 18 years vs. ≥18–64 years and age groups > 64 years vs. ≥18–64 years for Oseltamivir and Baloxavir Marboxil (Fig. 1 A-F).

For Oseltamivir (Fig. 1 A-C).

-In the age group < 18 years compared to ≥ 18–64 years, a significant safety signal was observed in nervous system disorders [ROR (95% CI), 1.26 (1.18–1.34) vs. 0.94 (0.89-1.00)].
-In the age group > 64 years compared to ≥ 18–64 years, more significant safety signals were observed in renal and urinary disorders [ROR (95% CI), 2.16 (1.91–2.44) vs. 0.83 (0.73–0.94)], metabolism and nutrition disorders [ROR (95% CI), 1.56 (1.36–1.79) vs. 0.94 (0.89-1.00)], and cardiac disorders [ROR (95% CI), 1.66 (1.48–1.88) vs. 0.76 (0.68–0.84)].

For Baloxavir Marboxil(Fig. 1 D-F).

-In the age group < 18 years compared to ≥ 18–64 years, a more significant safety signal was observed in general disorders and administration site reactions [ROR (95% CI), 1.69 (1.47–1.93) vs. 0.59 (0.48–0.74)].
-In the age group > 64 years compared to ≥ 18–64 years, more significant safety signals were observed in metabolism and nutrition disorders [ROR (95% CI), 2.80 (1.77–4.45) vs. 1.00 (0.61–1.64)], cardiac disorders [ROR (95% CI), 2.56 (1.59–4.12) vs. 0.72 (0.41–1.27)], musculoskeletal and connective tissue disorders [ROR (95% CI), 1.08 (0.69–1.69) vs. 0.43 (0.27–0.68)], and blood and lymphatic system disorders [ROR (95% CI), 1.52 (0.78–1.94) vs. 0.58 (0.29–1.17)].

### Gender-based stratified analysis of system organ class (SOC) signals

**Fig. 2:**
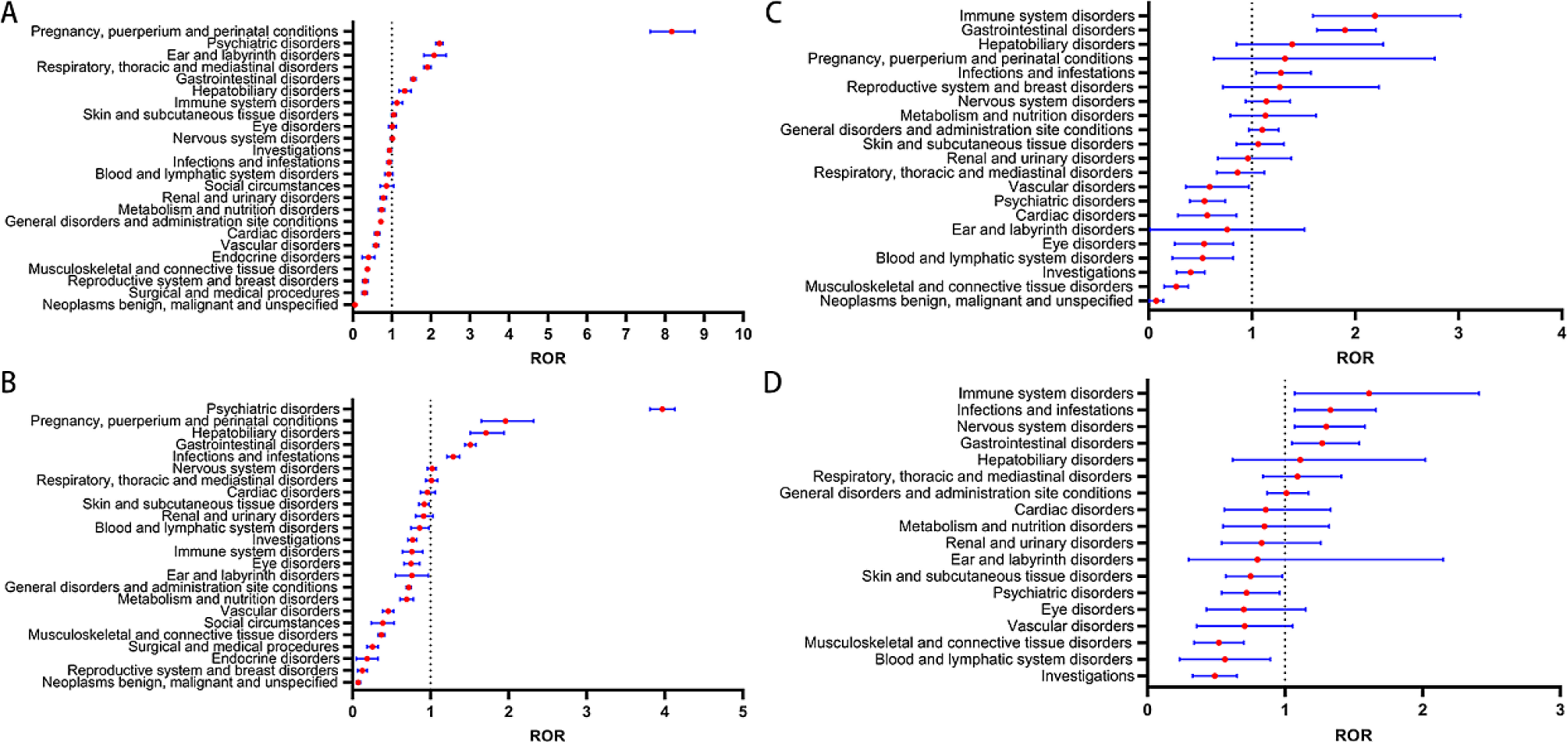
System Organ Class (SOC) Safety Signals in different sex groups. Figure 2 **A-B** represent safety signals for patients receiving oseltamivir treatment, with fig (**A**) for females and (**B**) for males; Fig. 2 **C-D** represents the safety signals of patients receiving treatment with baloxavir marboxil, with figures (**C**) for females and (**D**) for males. SOC stands for System Organ Class, and ROR stands for Reporting Odds Ratio

Differential analysis of SOC signal strength was conducted for male and female subgroups receiving Oseltamivir and Baloxavir Marboxil (Fig. 2 A-D).

For Oseltamivir (Fig. 2 A-B).

-In general, the number of reports and ROR were roughly similar between males and females.
-Compared to female patients, male patients showed a more significant safety signal in Infections and infestations [ROR (95% CI), 1.29 (1.21–1.37) vs. 0.93 (0.88–0.99)].
-Female patients exhibited more significant safety signals in Skin and subcutaneous tissue disorders [ROR (95% CI), 1.05 (0.99–1.11) vs. 0.92 (0.85–0.99)], Eye disorders [ROR (95% CI), 1.01 (0.92–1.11) vs. 0.75 (0.66–0.86)], and Ear and labyrinth disorders [ROR (95% CI), 2.08 (1.82–2.39) vs. 0.74 (0.56–0.98)].

For Baloxavir marboxil (Fig. 2 C-D).

-Similar to Oseltamivir, the number of reports and ROR were roughly similar between males and females.
- Compared to female patients, male patients showed a more significant safety signal in Respiratory, thoracic, and mediastinal disorders [ROR (95% CI), 1.09 (0.84–1.41) vs. 0.86 (0.66–1.12)].
-Female patients exhibited more significant safety signals compared to male patients in Skin and subcutaneous tissue disorders [ROR (95% CI), 1.06 (0.85–1.31) vs. 0.75 (0.57–0.98)], Reproductive system and breast disorders [ROR (95% CI), 1.27 (0.72–2.23) vs. 0], Pregnancy, puerperium, and perinatal conditions [ROR (95% CI), 1.32 (0.63–2.77) vs. 0], and Metabolism and nutrition disorders [ROR (95% CI), 1.13 (0.79–1.62) vs. 0.85 (0.55–1.32)].

### Disproportionality analysis of AEs in Oseltamivir

**Fig. 3:**
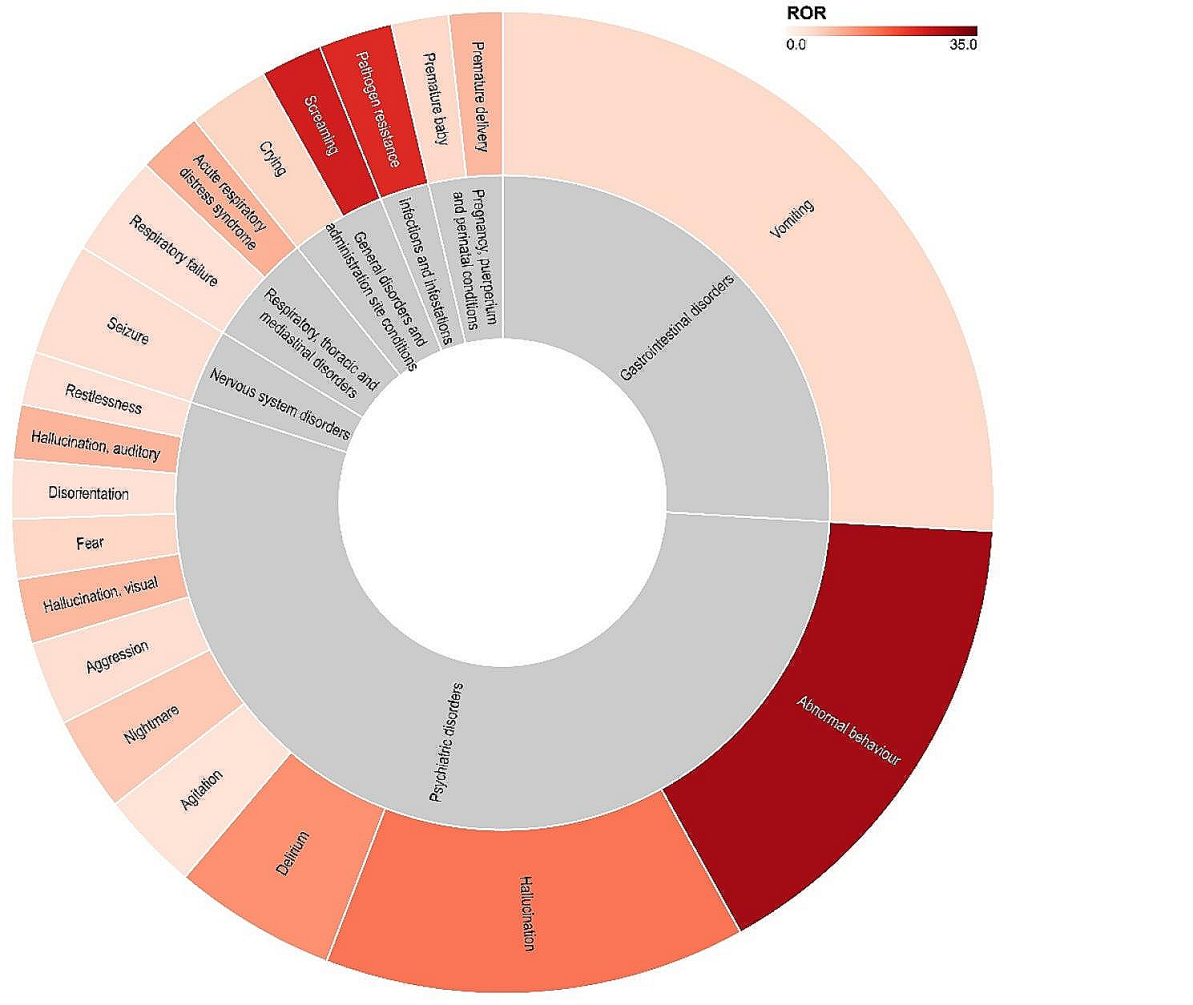
The Sunburst chart illustrates safety signals for adverse events (AE) in Oseltamivir. The outer ring represents individual AEs, while the inner ring represents the System Organ Class (SOC) of AEs. The size of each sector indicates the quantity of AEs, and the color gradient represents the Reporting Odds Ratio (ROR) values for the AEs. AE stands for Adverse Event, SOC stands for System Organ Class, and ROR stands for Reporting Odds Ratio

The Sunburst chart is utilized to illustrate the Reporting Odds Ratio (ROR) and report numbers for AEs (Top 20 in terms of report numbers) (Fig. 3). Among the identified AEs, the top 5 events with the highest ROR values are Appendicolith, acne infantile, Acute macular neuroretinopathy, proctitis, and purpura senile [ROR (95% CI) values are 459.53 (340.88-619.47)], 368.65 (118.89-1143.09)], 294.92 (97.88-888.64)], 245.74 (101.47-595.31)], and 154.02 (81.96-289.43)], respectively. The top 5 ranked AEs by the number of reports are vomiting, abnormal behavior, hallucination, delirium, and seizures (*n* = 1495, 923, 800, 309, 215). For DMEs, signals are elevated for hepatitis fulminant, anaphylactic shock, ventricular fibrillation, toxic epidermal necrolysis, autoimmune hemolytic anemia, erythema multiforme, Stevens-Johnson syndrome, anaphylactoid reaction, deafness transitory, and immune thrombocytopenia [ROR (95% CI) are 12.12 (8.30-17.72), *n* = 27; 7.92 (2.54–24.65), *n* = 3; 7.68 (6.01–9.83), *n* = 64; 7.21 (5.74–9.05), *n* = 75; 6.65 (2.76–16.01), *n* = 5; 5.67 (4.14–7.77), *n* = 39; 5.36 (4.36–6.59), *n* = 90; 4.58 (2.71–7.75), *n* = 14; 4.46 (1.43–13.86), *n* = 3; 3.45 (1.43–8.30), *n* = 5].

### Disproportionality analysis of AEs in Baloxavir marboxil

**Fig. 4:**
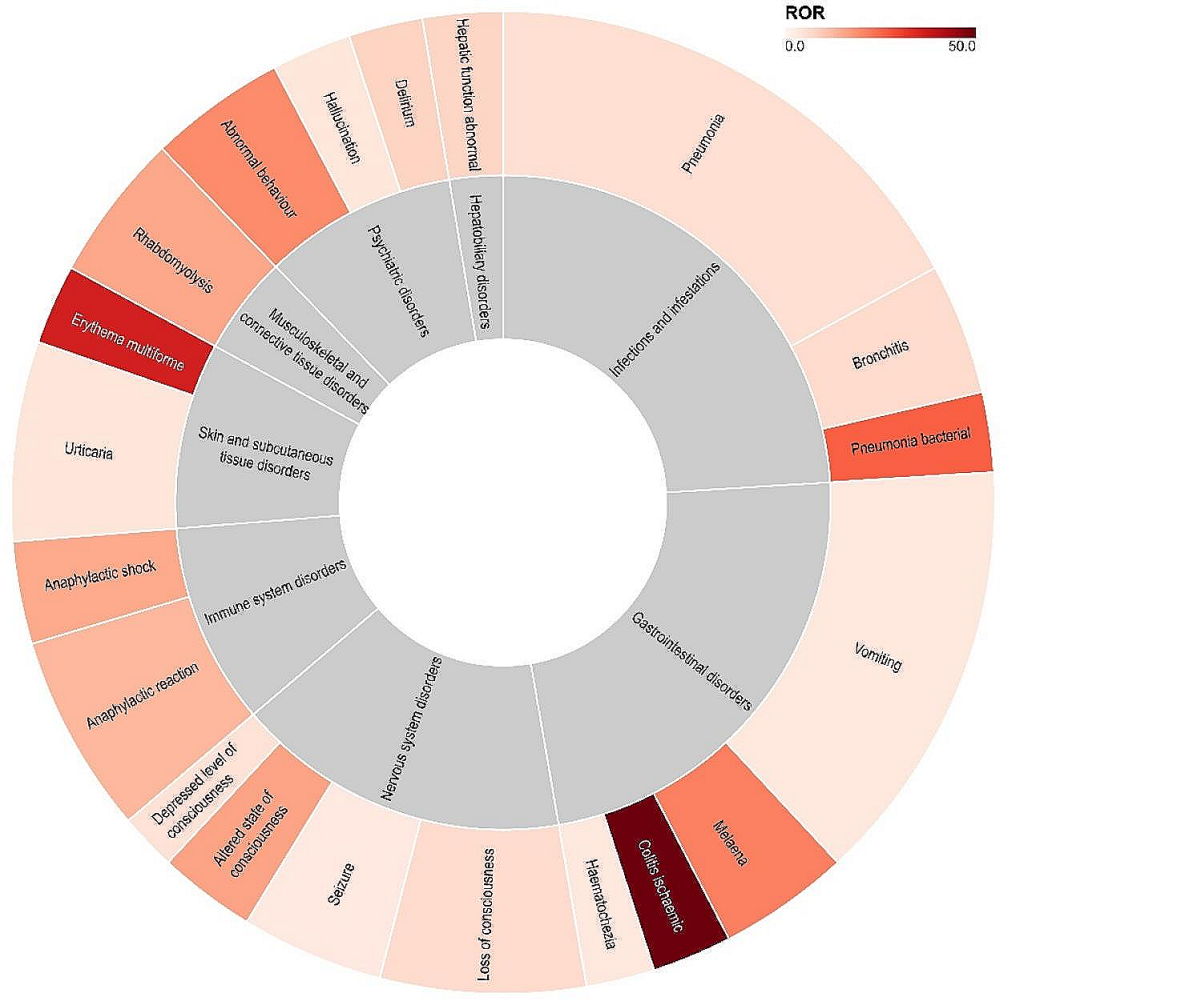
The Sunburst chart illustrates the safety signals of adverse events (AE) in Baloxavir Marboxil. The outer circle represents individual AEs, and the inner circle represents the System Organ Class (SOC) of AEs. The size of each sector indicates the number of occurrences of the AE, and the color intensity represents the Reporting Odds Ratio (ROR) values for each AE. AE stands for Adverse Event, SOC stands for System Organ Class, and ROR stands for Reporting Odds Ratio

The Sunburst chart is utilized to depict the Reporting Odds Ratio (ROR) and the number of reports (top 20) for AEs in Baloxavir Marboxil (Fig. 4). Among the identified AEs, the top 5 with the highest ROR values are delirium febrile, febrile convulsion, colitis ischemic, erythema multiforme, and pneumonia bacterial [ROR (95% CI) are 1698.97 (734.33-3930.81), 123.76 (51.02-300.19), 49.52 (29.23–83.88), 36.22 (21.39–61.32), and 26.21 (15.49–44.35) respectively]. The top 5 AEs by report count are pneumonia, vomiting, loss of consciousness, allergic reaction, and urticaria (*n* = 91, 75, 36, 35, 35). For DMEs, erythema multiforme, rhabdomyolysis, anaphylactic shock, allergic reaction, and Stevens-Johnson syndrome are highlighted [ROR (95% CI) are 36.22 (21.39–61.32), *n* = 14; 15.50 (10.53–22.80), *n* = 26; 15.14 (9.52–24.08), *n* = 18; 12.87 (9.22–17.96), *n* = 35, and 3.86 (1.24–11.97), *n* = 3].

### Comparison of significant safety signals between Baloxavir marboxil and Oseltamivir

**Fig. 5:**
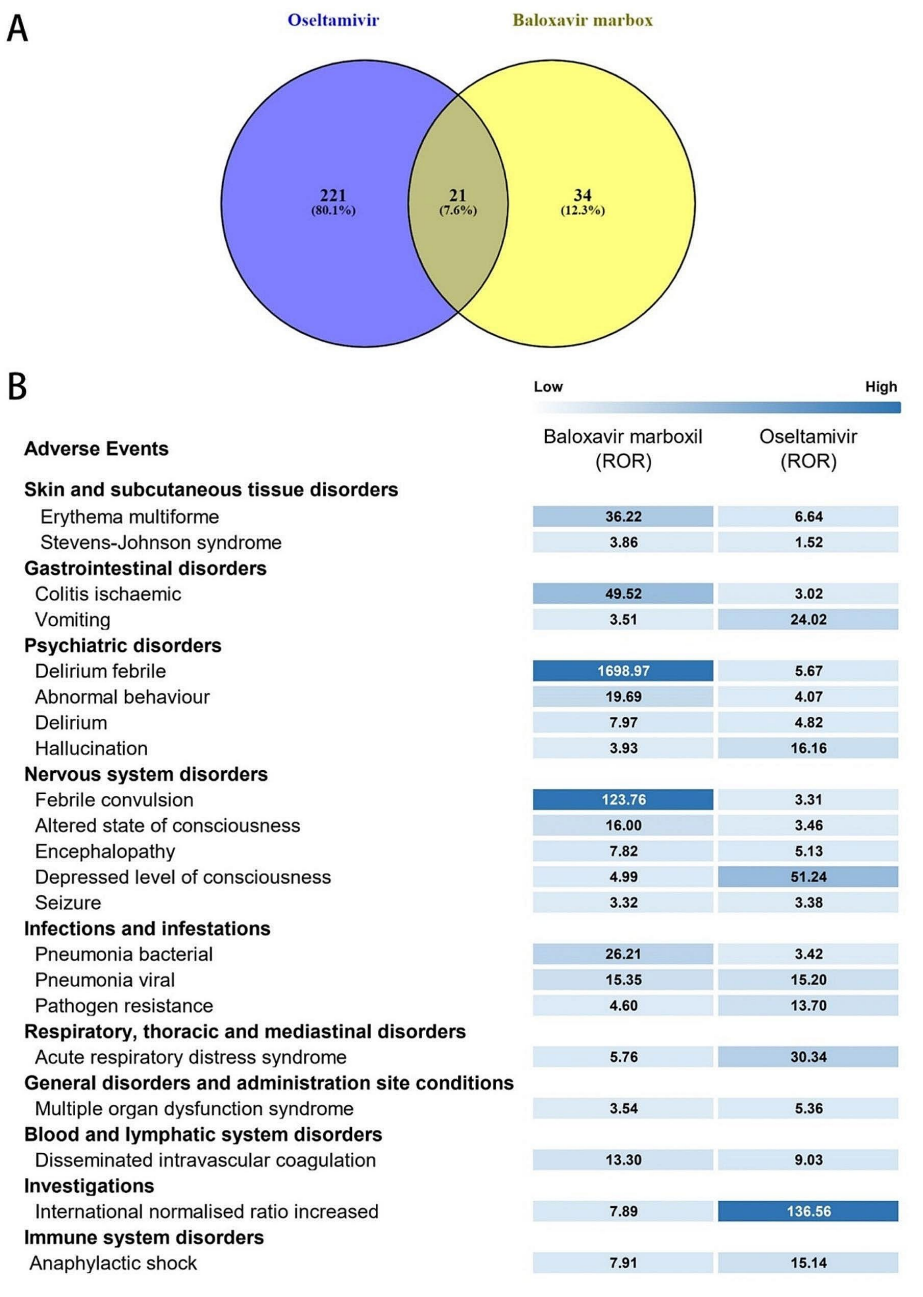
Comparative analysis of significant safety signals between Oseltamivir and Baloxavir marboxil. (**A**) The Venn analysis between Oseltamivir and Baloxavir marboxil. (**B**) The common safety signals of the two drugs

The Venn analysis (Fig. 5A-B) reveals Common AEs for both drugs include those related to the skin, gastrointestinal system, mental health, nervous system, infections, respiratory system, blood, and immune system diseases, as depicted in Fig. 5B. Specific AEs for which Oseltamivir was significant included appendiceal calculus [ROR (95% CI), 459.53 (340.88 to 619.47)], acne infantile [ROR (95% CI), 368.65 (118.89 to 1143.09)], acute macular ectopia retinopathy [ROR (95% CI), 294.92 (97.88 to 888.64)], proctitis [ROR (95% CI), 245.74 (101.47 to 595.31)], and purpura senile [ROR (95% CI), 154.02 (81.96 to 289.43)]. Furthermore, DMEs for Oseltamivir include hepatitis fulminant [ROR (95% CI): 12.12 (8.30-17.72), *n* = 27], ventricular fibrillation [ROR (95% CI): 7.68 (6.01–9.83), *n* = 64], toxic epidermal necrolysis [ROR (95% CI): 7.21 (5.74–9.05), *n* = 75], among others. For Baloxavir Marboxil, significantly specific AEs include Melaena [ROR (95% CI): 21.34 (14.15–32.18), *n* = 23]; Cystitis haemorrhagic [ROR (95% CI): 20.22 (7.57-54.00), *n* = 4]; ileus paralytic [ROR (95% CI): 18.57 (5.98–57.71), *n* = 3]; Haemorrhagic diathesis [ROR (95% CI): 16.86 (5.43–52.40), *n* = 3] Rhabdomyolysis [ROR (95% CI): 15.50 (10.53–22.80), *n* = 26]; Additionally, DMEs for Baloxavir Marboxil also include rhabdomyolysis [ROR (95% CI): 15.50 (10.53–22.80), *n* = 26]. Reveals a comparison of significant safety signals between Baloxavir Marboxil and Oseltamivir, identifying 21 common AEs. Additionally, Oseltamivir has 221 AEs specific to it, while Baloxavir Marboxil has 34 specific AEs, as shown in Fig. 5 A.

## Discussion

To our knowledge, we are the first study the AEs of Oseltamivir and Baloxavir marboxil were comprehensively evaluated and compared using a vast real-world AE reporting database. The primary objective was to provide valuable insights for clinical drug selection. The key findings are summarized below:

Firstly, Oseltamivir exhibited gastrointestinal diseases, including nausea and vomiting, as the primary observed adverse reactions, consistent with clinical trial results [9, 10]. Unprecedented DMEs were identified, such as fulminant hepatitis, autoimmune hemolytic anemia, transient deafness, and immune thrombocytopenia. Fulminant hepatic failure induced by Oseltamivir is a DME, though the definite correlation with clinically significant liver damage remains unclear. Increasing evidence suggests potential toxic liver injury during Oseltamivir treatment [11]. The mechanism of Oseltamivir-induced liver damage is not well understood, with some studies proposing a link to the inhibition of endogenous neurotransmitter enzyme activity [12]. Lower hepatic carboxylesterase and P-glycoprotein activity in infants and immature animals may contribute to increased Oseltamivir concentrations, leading to toxic effects [13]. Animal toxicity tests support clinical evidence of liver disease [14–16]. Despite challenges in establishing a clear causal relationship between Oseltamivir and liver damage, given the severity of AEs, vigilant monitoring of liver function abnormalities is advisable, especially in patients with underlying liver diseases. Among others, support clinical evidence of liver disease, such as oxidative stress in the liver, pathological changes in liver tissue, and acute toxicity. The ROR value for hepatitis fulminant is the highest among DMEs identified in this survey. Due to the short exposure time to Oseltamivir and the potential hepatotoxicity of concomitant use of antipyretic analgesics, antibiotics, etc., the process of CD8 + T cell infiltration into the liver during influenza infection itself can lead to clinically significant hepatitis [17]. However, severe liver dysfunction and hepatitis fulminant associated with influenza virus infection are rarely reported in children [18] and adult patients [19]. Based on the above, it is challenging to determine the causal relationship between Oseltamivir and liver damage. Still, considering the DMEs, it is advisable to monitor liver function abnormalities promptly and discontinue or switch to alternative influenza treatment during medication, especially in patients with underlying liver diseases. Monitoring liver-related indicators is essential and requires special attention. Regarding autoimmune hemolytic anemia and immune thrombocytopenia, it suggests that Oseltamivir may have an impact on the immune system.

Influenza itself might be associated with thrombocytopenia or aberrant coagulation, especially in severe cases with systemic inflammatory response syndrome, including avian influenza [20–23]. The mechanism by which Oseltamivir induces thrombocytopenia is currently unclear, and whether Oseltamivir affects platelet clearance through rapid clearance by liver cells and subsequent sialic acid residue cleavage on the platelet surface is not well understood [24]. Healthcare professionals should be vigilant about the risk of thrombocytopenia associated with Oseltamivir use, especially when used concomitantly with drugs that may increase the risk of thrombocytopenia. Monitoring patients’ platelet counts is recommended, and healthcare providers should be contacted immediately if symptoms of thrombocytopenia occur. Although Deafness transitory is a newly discovered AE associated with Oseltamivir, there are literature reports [25] that deafness is an extremely rare complication caused by influenza A virus. Therefore, further exploration is needed to determine the correlation between deafness and Oseltamivir.

Among the reported AEs related to Oseltamivir, abnormal behavior ranked second in terms of quantity. These spontaneous reports of clinical observations of psychiatric symptoms (especially cases of abnormal behavior leading to sudden death or fatal consequences, often occurring after sleep) are consistent with some prospective cohort studies [26–30], systematic reviews of cohort studies [31], and systematic reviews of randomized controlled trials [32]. Possible explanations for the occurrence of neuro-psychiatric adverse events in patients taking Oseltamivir include an increase in plasma and intracranial concentrations of unchanged Oseltamivir due to decreased activity of hepatic carboxylesterase and P-glycoprotein. Both enzymes and P-glycoprotein activity are influenced by pro-inflammatory cytokines during the acute phase of influenza infection and immaturity [13]. Inhibition of monoamine oxidase A is another mechanism for abnormal behavior [13]. Additionally, inhibiting endogenous neurotransmitter enzymes in patients may be one of the potential mechanisms for inducing delayed abnormal behavior [12]. Suzuki et al. [33] reported that Oseltamivir liquefies serum glycolipids, and this modified glycolipid induces jumping behavior by stimulating dopamine D2 receptors. They believe that this mechanism may be related to abnormal behavior in some children taking Oseltamivir. In Japan, the use of Oseltamivir is generally prohibited for individuals aged 10–19 because of concerns about abnormal behavior. Our observations also found a more significant safety signal in the nervous system diseases when comparing age < 18 with age ≥ 18–64. Consistent with the report in reference [34]. There is also increasing evidence of an association between them [13]. Therefore, clinical doctors are advised to pay close attention to abnormal behavior that may occur during Oseltamivir treatment or prophylaxis. Special attention should be given to patients aged 10–19 or those with a history of neurological and psychiatric diseases.

Secondly, for Baloxavir marboxil, this study observed that the main adverse reactions were gastrointestinal diseases, including nausea, diarrhea, etc., which is consistent with the adverse reactions of Baloxavir marboxil in clinical trials [35]. It is noteworthy that some studies [36] have shown that among the most frequently reported AEs (≥ 5%), Baloxavir marboxil has a lower incidence of vomiting (5% vs. 18%) and diarrhea (5% vs. 0%) compared to Oseltamivir. Diarrhea is one of the special AEs caused by Baloxavir marboxil and has a significant safety signal, possibly due to the excretion of Baloxavir marboxil and its active metabolites in the feces, chelating metal ions in the food in the intestines, and increasing osmotic pressure [37], leading to diarrhea. Baloxavir marboxil discovered new DMEs, including rhabdomyolysis. Baloxavir marboxil showed a significant signal of rhabdomyolysis in diseases of the musculoskeletal and connective tissue. In the safety signal of Baloxavir marboxil, rhabdomyolysis was found, along with related safety signals such as elevated blood creatine phosphokinase, renal failure, and even multiple organ dysfunction, disseminated intravascular coagulation, suggesting that rhabdomyolysis may occur after taking the drug. However, the specific mechanism is currently unclear and requires further clinical research for analysis. In addition, influenza is also a high-risk factor for rhabdomyolysis, so whether it is drug-induced requires further evaluation. However, for influenza patients taking statins and other drugs, extra attention should be paid to the occurrence of rhabdomyolysis when using Baloxavir marboxil, and clinical monitoring of blood creatine phosphokinase, renal function, coagulation indicators, etc., is necessary if clinical manifestations occur. In addition, melaena, cystitis haemorrhagic, haemorrhagic diathesis are all related to bleeding, and there are also relevant literature reports [34]. However, the mechanism is not yet clear. If patients with haemorrhagic diathesis use Baloxavir marboxil, close attention should be paid to coagulation indicators and bleeding conditions.

Thirdly, both Oseltamivir and Baloxavir marboxil warrant vigilance for DMEs such as Stevens-Johnson syndrome, abnormal behavior, anaphylactic shock, and delirium. For children, especially those aged 5–11 with pre-existing diarrhea, Oseltamivir may be considered, and if vomiting occurs, Baloxavir marboxil may be a better choice. For children under 18, due to the risk of abnormal behavior associated with Oseltamivir, especially in patients with neurological or psychiatric underlying diseases, although Baloxavir marboxil also has neurological and psychiatric disease-related AEs, there are no significant safety signals in patients under 18 years old. Perhaps for patients in this age group, Baloxavir marboxil may be one of the preferred options. For elderly patients aged 65 and above, with underlying Metabolism and nutrition disorders and heart diseases, both Oseltamivir and Baloxavir marboxil need close attention. For patients with underlying liver diseases, due to the potential risk of fulminant hepatitis, Baloxavir marboxil may be a preferred option. For influenza patients taking statins and other drugs, as well as elderly patients aged 65 and above, due to the potential risk of rhabdomyolysis when using Baloxavir marboxil, Oseltamivir may be a preferred option. For elderly patients with underlying kidney and urinary system diseases, Baloxavir marboxil may be a potential preferred option. In summary, considerations for drug selection based on age, underlying conditions, and specific risks are outlined.

However, it is crucial to note the limitations of the study, First, the observed data only indicate that the increased risk is associated with a particular drug, not conclusively identify adverse reactions caused by the drug. Only a statistical association was proved, and the inevitable causal relationship still needs to be confirmed by further clinical studies. Therefore, future clinical studies should consider using the Naranjo adverse drug reaction probability scale to determine whether there is a causal relationship between AEs and the drugs under investigation [38]. Second, it is important to note that bias may exist, but it is unlikely to be completely present from the study [5]. The observed safety signal may be influenced by relevant factors such as loss of data, disease complications, comorbidities, and drug interactions. Third, due to the different marketing time of the two drugs, the sample size of the two drugs in this study is quite different, which may be biased. In addition, baloxavir marboxil is poorly reported by professionals, which may lead to the underreporting of some minor adverse events. Fourth, although the study included a large sample of patients with influenza, the safety profile assessment of both drugs may remain incomplete due to flaws in the database itself. This is because the FAERS database, as a spontaneous reporting system, registers only reported cases, not all cases. Therefore, even if the total number of patient groups administered could be determined, the incidence of ae could not be accurately estimated [39]. Fifth, we chose to present raw data without merging similar ae to avoid introducing elimination of human bias. Sixth, despite efforts to compare the safety of the signals between the two drugs, it is important to recognize that these signals may be confused with influenza symptoms, disease progression, etc.

## Conclusion

This study offers crucial safety insights for guiding drug selection in Seasonal influenza therapy. Key findings highlight the need for vigilant monitoring of fulminant hepatitis during Oseltamivir treatment, especially in patients with liver-related diseases. Oseltamivir’s potential to induce abnormal behavior, particularly in adolescents, underscores the importance of careful monitoring. Baloxavir Marboxil, with lower hepatic toxicity, emerges as a promising alternative for patients with liver diseases. During its use, close attention to the possibility of rhabdomyolysis is crucial, necessitating timely monitoring of relevant indicators for patients with clinical manifestations. This research underscores the importance of tailored drug choices based on individual patient characteristics and health conditions. Since no causal association is established for various safety signals and given the possible confounding factors, This research all the potential predictions are subject to findings of active targeted pharmacovigilance studies in influenza patients.

## Data Availability

The data is openly available in the FDA Adverse Event Reporting System Public Dashboard at: https://fis.fda.gov/extensions/FPD-QDE-FAERS/FPD-QDE-FAERS.html.
